# A Challenging Case of Multifocal *Mycobacterium marinum* Osteoarticular Infection in a Patient with Anorexia Nervosa

**DOI:** 10.1155/2015/963138

**Published:** 2015-06-07

**Authors:** Katie Sharff, Zaw Min, Nitin Bhanot

**Affiliations:** ^1^Kaiser Permanente Infectious Disease Department, 9900 SE Sunnyside Road, Clackamas, OR 97015-977, USA; ^2^Division of Infectious Disease, Department of Medicine, Allegheny General Hospital, Allegheny Health Network, 420 East North Avenue, Pittsburgh, PA 15212, USA

## Abstract

Disseminated infection due to *Mycobacterium marinum* is rare but has been described in immunosuppressed and transplant recipients. We describe a case of multifocal osteoarticular involvement by this pathogen in a patient with anorexia nervosa. Serial surgical debridements and a prolonged course of antimicrobial therapy including intravenous amikacin, imipenem, and oral ethambutol and azithromycin were needed to treat the infection. Cell mediated immune deficits related to the patient's anorexia nervosa predisposed her to a severe infection with *M. marinum.* Aggressive surgical intervention in conjunction with multiple antimicrobial agents helped cure the infection.

## 1. Introduction

*Mycobacterium marinum* is a nontuberculous mycobacterium that is found in fresh and salt water, including swimming pools, fish tanks, and marine environments [1].* M. marinum* causes cutaneous disease and usually appears as a solitary papule on an extremity that can progress to ulceration and scar formation. Although most patients present with a single lesion, lymphatic or local spread has been described [2]. Disseminated disease is rare but has been reported in immunosuppressed and transplant patients [3]. Herein, we describe a case of disseminated* M. marinum* disease involving left thigh muscle and bilateral knees and ankles in a patient with severe anorexia nervosa. The case highlights the immune defects in anorexia that likely predisposed our patient to this infection and underscores the need for surgical intervention to aid in treatment of this chronic and debilitating infection in severe cases.

## 2. Case

**Figure 1:**
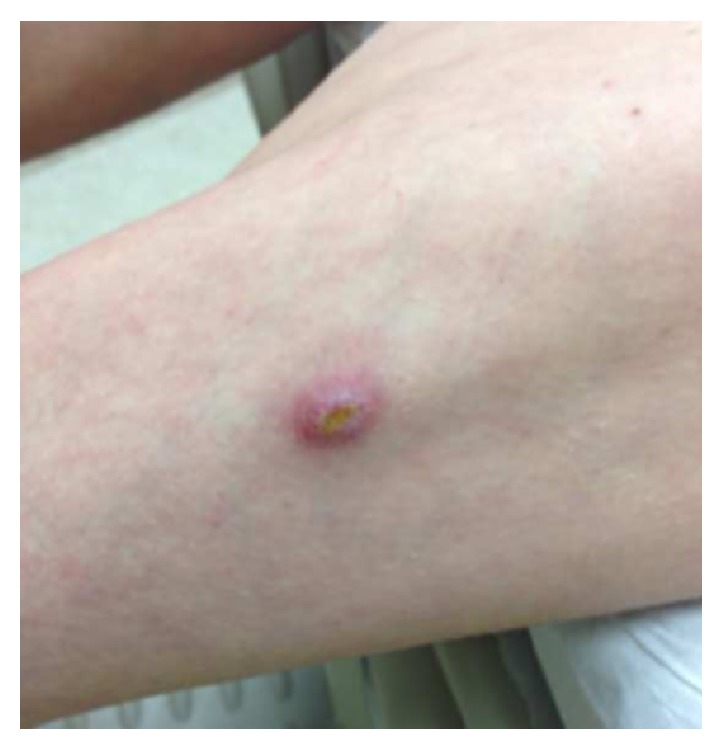
Initial nodule on the thigh that regressed to a small ulcer.

A 29-year-old pleasant female presented with bilateral knee swelling of 2-3-week duration. The patient had spontaneously developed a left thigh cutaneous nodule approximately 6 months prior to presentation. This was treated with oral trimethoprim/sulfamethoxazole (TMP/SMX) for suspected methicillin resistant* Staphylococcus aureus* infection and the nodule regressed to a small ulcer but never completely resolved (Figure 1). The patient's past medical history was significant for anorexia nervosa (body mass index: 14.9 kg/m^2^) that was complicated by recurrent episodes of hypoglycemia, seizures, and a history of a cardiac arrest related to malnutrition. She had been hospitalized many times for inpatient treatment of anorexia nervosa. She denied alcohol, tobacco, or illicit drug use. She cared for a cat, fish, and birds at home but denied any bites or scratches from these animals.

On examination, both knees were warm with bilateral effusions. She had limited range of motion of both knee joints with some tenderness on palpation of the medial femoral condyles. Laboratory investigations revealed normal chemistry and liver panel, a white blood cell (WBC) count of 2600 cells/mm^3^, hematocrit of 24.1%, and platelets of 132,000 cells/mm^3^. Her blood cultures and HIV serology were negative. The patient underwent aspiration of the knees with approximately 30 mL of purulent material removed from each knee. Joint fluid demonstrated a leukocyte count of 20,000 cells/mm^3^ with 92% neutrophils. Gram stain was negative; however the Ziehl-Neelsen stain (acid-fast Bacilli (AFB) stain) from the right knee aspirate was positive. After 20 days, cultures on Lowenstein-Jensen medium from both knee aspirates grew* Mycobacterium marinum*. This organism was confirmed by 16S ribosomal DNA sequencing.

**Figure 2:**
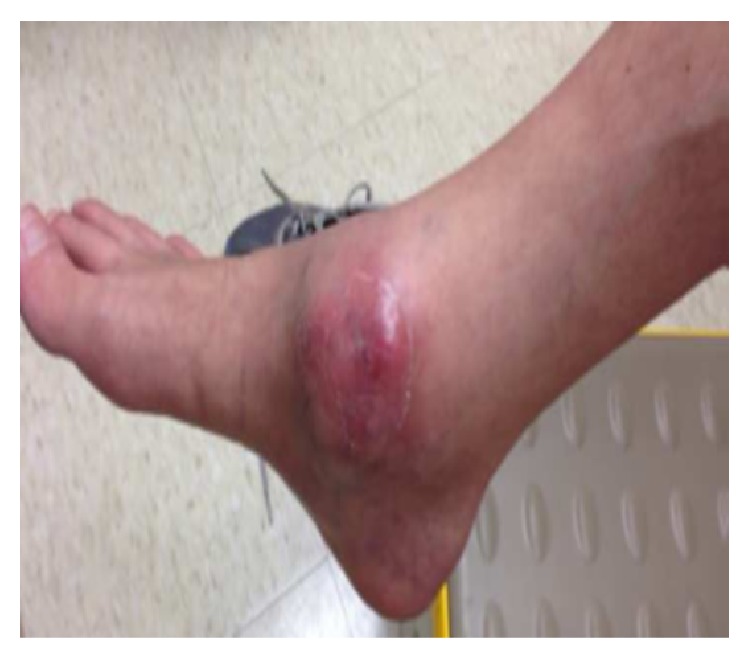
Swelling over the right ankle.

**Figure 3:**
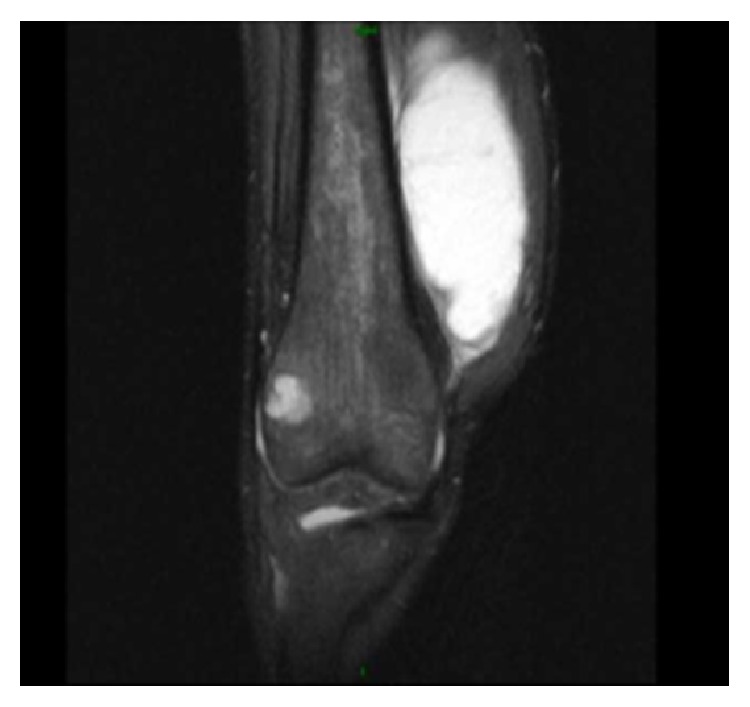
Abscesses around the right knee joint.

**Figure 4:**
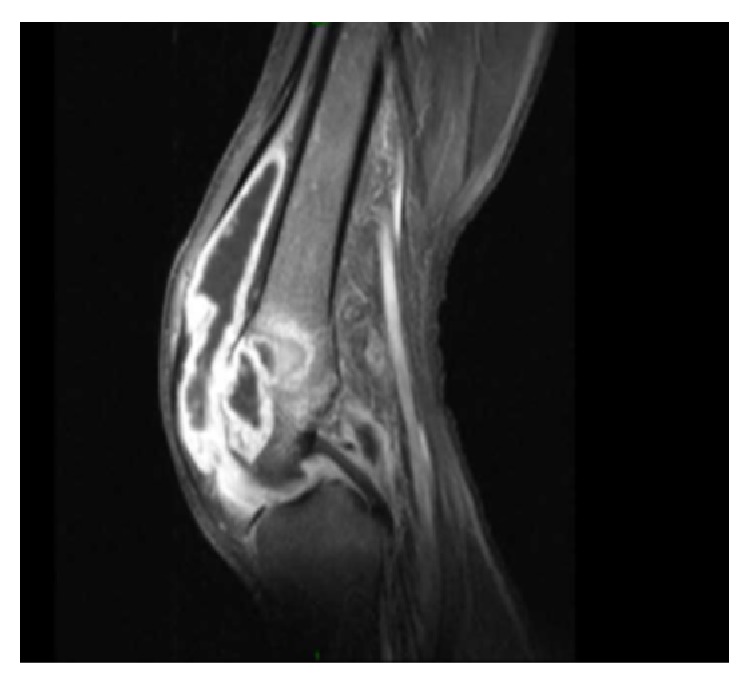
Abscesses around the left knee joint.

The patient was started on therapy with azithromycin, rifampin, and ethambutol. She tolerated the triple antibiotic therapy but had ongoing pain, swelling, and effusions in her knees without any significant relief. About a month into therapy, she additionally developed swelling, erythema, and pain in her ankles (Figure 2). Magnetic resonance imaging (MRI) of her knees was performed. The right knee MRI showed lateral femoral condyle and medial tibial plateau abscesses with an associated 8.3 cm × 4.1 cm abscess within the vastus medialis muscle (Figure 3). The MRI of her left knee revealed septic arthritis, multifocal intraosseous abscesses, and distal femoral and proximal tibial osteomyelitis (Figure 4). The patient was taken to the operating room and underwent arthrotomy and debridement of both knees and ankles.

**Figure 5:**
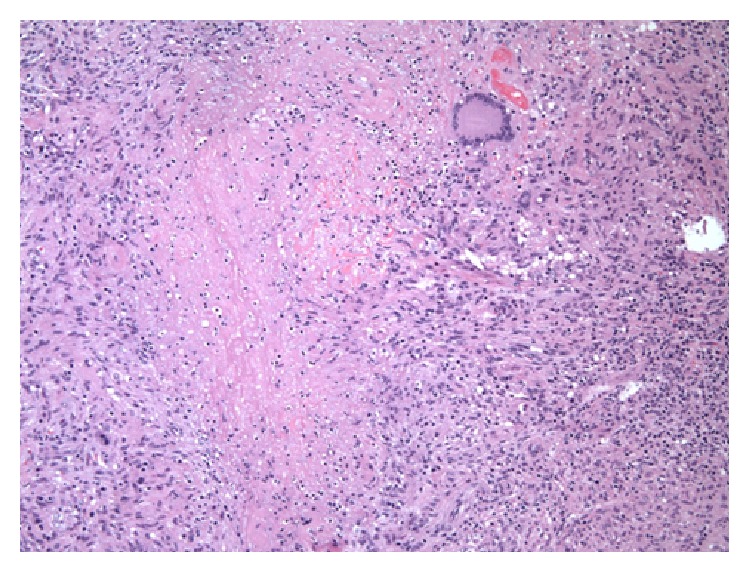
Palisading granulomas from the thigh and ankle regions.

The patient demonstrated initial clinical improvement following surgery; however, she developed recurrent abscess formation over the left thigh and in her right ankle requiring excision of the left thigh abscess and a repeat irrigation and debridement of the right ankle. The AFB stain of the right ankle and left thigh tissues was negative. The histopathology of tissues from the thigh and ankle demonstrated necrotizing palisading granulomas (Figure 5). The right ankle mycobacterial tissue culture eventually grew* M. marinum*. The isolate was sent for sensitivity testing and demonstrated* in vitro* resistance to rifampin, which was stopped. She was continued on ethambutol and azithromycin. Given her extensive disease, TMP/SMX and moxifloxacin were added to her regimen with careful EKG monitoring.

**Figure 6:**
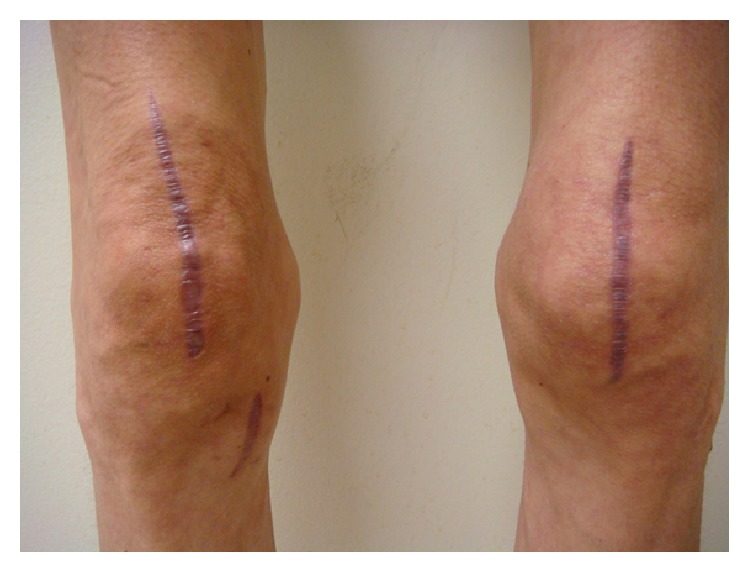
Healed lesions of the knees.

**Figure 7:**
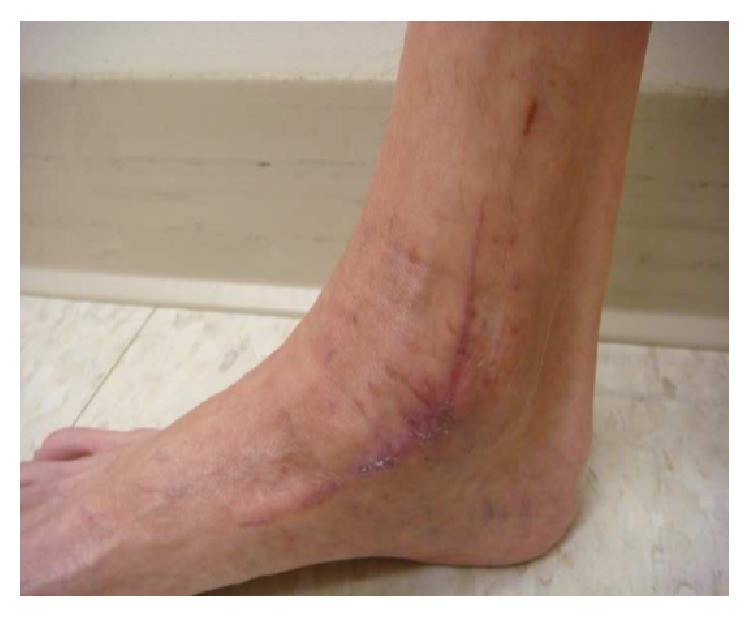
Healed lesion in the right ankle.

**Figure 8:**
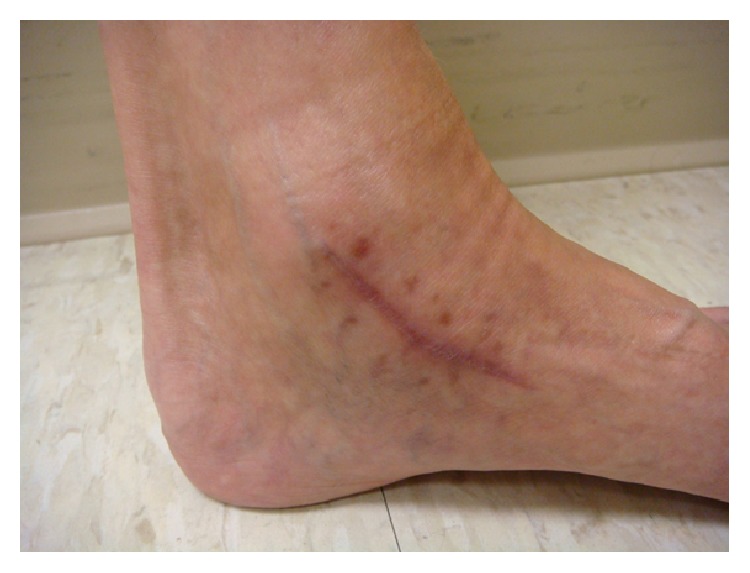
Healed lesion in the left ankle.

Her clinical course was complicated by pancytopenia which was thought to be secondary to antibiotics or bone marrow suppression from progressive mycobacterial infection. Thus, the antibiotic regimen was modified. TMP/SMX and moxifloxacin were stopped; intravenous amikacin (25 mg/kg/day) three days a week and intravenous imipenem (500 mg every 8 hours) were added to ethambutol (800 mg every 24 hours) and azithromycin (250 mg every 24 hours). Amikacin drug levels were intermittently checked and were in the range of 0.5–0.6 mcg/mL. The plan was a treatment for a 12-week course with 4-drug therapy followed by additional 12 weeks with a single agent, azithromycin. The gene study on the interferon-gamma receptors (IFNGR) 1 and 2 did not demonstrate mutations or deficiency of IFNGR-1 and IFNGR-2, which could have predisposed the patient to mycobacterial infection. The patient received nocturnal intravenous total parenteral nutrition (TPN) in an attempt to boost the host immune response to mycobacterial infection. Her family provided excellent social and emotional support during the course of therapy. At the 10th week of the 4-drug therapy, the patient developed nausea, lethargy, thrombocytopenia, and elevation of liver enzymes. A concern for drug-induced hepatocellular hepatitis and thrombocytopenia prompted a change to azithromycin monotherapy with a rapid resolution of her symptoms and laboratory abnormalities. Azithromycin was continued as monotherapy for 14 weeks uneventfully. The patient completed a total of 6 months of therapy (10 weeks of combined quadruple drug therapy plus 14 weeks of azithromycin monotherapy). A complete resolution of swelling in her knees and ankles (Figures 6–8) and normalization of inflammatory markers was achieved at the end of treatment. At the 3-month follow-up after completion of antimicrobial therapy, the patient did not reveal signs and symptoms suggestive of a relapse of the infection. She continues to be on TPN and has gained 7 pounds since the initiation of this form of supplemental nutrition. She is on sertraline 100 mg daily for anorexia and has scheduled follow-ups with the infectious disease service for close clinical surveillance.

## 3. Discussion

*M. marinum* is pigmented, slowly growing organism that has been described as the cause of swimming pool granuloma or fish tank granuloma [1]. The organism is introduced into the skin through open abrasions while cleaning a fish tank or by scratches or puncture wounds from saltwater fish [1, 4]. We were, however, unable to establish the route of inoculation of the microbe in our patient's lower extremities. Diagnosis is made from a combination of histological examination and culture [5].

Lesions usually appear as a solitary papule and infection is often localized to the upper limb. Sporotrichoid* M. marinum* infection occurs in about 20% of cases and involves spread of infection from the primary nodule to regional lymph nodes [2]. Upper extremity tenosynovitis, osteomyelitis, and septic arthritis have been reported but are usually localized to one joint [6]. Disseminated* M. marinum* infections have been described but usually occur in the immunocompromised host [3]. Cases of multiple skin lesions including the face, upper limbs, and legs have been described in patients with history of liver transplantation [7]. Osteoarticular infection secondary to* M. marinum* was limited to skin and soft tissues and joints of upper extremities [8, 9]. Bilateral infection of knees and ankles septic arthritis and osteomyelitis from* M. marinum*, as in our patient, is a rare occurrence.

Clinical observations demonstrate that immunodeficiency is dependent on the severity of emaciation in anorexia nervosa [10]. Cellular immunity is generally preserved until weight reaches less than 60% of ideal body weight [11]. Restrictive anorexia nervosa has been shown to affect cell-mediated immunity with depletion in leukocyte, lymphocyte, and T-cell counts. Furthermore, patients with anorexia nervosa have elevated levels of proinflammatory cytokines such as interleukin-6 (IL-6) and tumor necrosis factor- (TNF-) alpha [10]. However, it has been suggested that, in the setting of infection, those cells may respond poorly to further stimulation and thereby have an impaired capacity to mount an acute-phase response to infection [10]. Another important primary immune disorder associated with increased susceptibility to mycobacterial infection is IFNGR-1 and IFNGR-2 deficiencies [12]. It was tested in our patient and did not show representative mutations in genes that code those receptors. A handful of cases of* Mycobacterium tuberculosis* and* Mycobacterium szulgai* pulmonary infections have been described in anorexic patients [13, 14]. Our patient's anorexia nervosa and associated immune dysregulation likely predisposed her to disseminated infection with* M. marinum*. To the best of our knowledge, it is the first reported case of disseminated* M. marinum* osteoarticular infection in a patient with anorexia nervosa.

There are no comparative trials evaluating treatment for* M. marinum*. Routine susceptibility testing is not recommended and is reserved for cases of treatment failure [5]. By standard testing, isolates are usually susceptible to rifampin, rifabutin, ethambutol, clarithromycin, and sulfonamides. General approach is treatment with 2 active agents until 1-2 months after resolution of symptoms [15]. The role of surgery is not well established but is recommended for deep infections or infections involving closed spaces [5]. In this instance, surgical intervention in the form of serial debridements was of paramount importance to reduce the high mycobacterial burden at multiple osteoarticular sites and aided in penetration of the antimicrobials at the site of infection. Given the severe progressive infection seen in our patient, we elected to use four active agents and utilized parenteral therapy for improved serum and tissue drug concentrations. We encountered adverse effects from antimicrobials during treatment of this infection for which no standardized guidelines exist. This was an ongoing therapeutic challenge. The case illustrates the need for susceptibility testing and repeated surgical intervention in cases where clinical improvement does not occur. It may also provide other clinicians with the choice of antimicrobial armamentarium in similar* M. marinum* infections.

## 4. Conclusion

Decreased cell-mediated immunity secondary to the patient's severe anorexia nervosa most likely predisposed her to this disseminated* M. marinum* osteoarticular infection. A multidisciplinary collaboration including aggressive surgical debridement, a combination of multiple antimicrobial agents, close clinical and blood work surveillance, and nutritional and family support played a pivotal and critical role in successful therapy for our patient.
